# Parental alcohol use and adolescent school adjustment in the general population: Results from the HUNT study

**DOI:** 10.1186/1471-2458-11-706

**Published:** 2011-09-19

**Authors:** Fartein A Torvik, Kamilla Rognmo, Helga Ask, Espen Røysamb, Kristian Tambs

**Affiliations:** 1 Division of Mental Health, Norwegian Institute of Public Health, P.O. Box 4404 Nydalen, 0403 Oslo, Norway; 2 Department of Psychology, University of Oslo, P.O. Box 1094 Blindern, 0317 Oslo, Norway

## Abstract

**Background:**

This study investigates the relationship between parental drinking and school adjustment in a total population sample of adolescents, with independent reports from mothers, fathers, and adolescents. As a group, children of alcohol abusers have previously been found to exhibit lowered academic achievement. However, few studies address which parts of school adjustment that may be impaired. Both a genetic approach and social strains predict elevated problem scores in these children. Previous research has had limitations such as only recruiting cases from clinics, relying on single responders for all measures, or incomplete control for comorbid psychopathology. The specific effects of maternal and paternal alcohol use are also understudied.

**Methods:**

In a Norwegian county, 88% of the population aged 13-19 years participated in a health survey (N = 8984). Among other variables, adolescents reported on four dimensions of school adjustment, while mothers and fathers reported their own drinking behaviour. Mental distress and other control variables were adjusted for. Multivariate analysis including generalized estimation equations was applied to investigate associations.

**Results:**

Compared to children of light drinkers, children of alcohol abusers had moderately elevated attention and conduct problem scores. Maternal alcohol abuse was particularly predictive of such problems. Children of abstainers did significantly better than children of light drinkers. Controlling for adolescent mental distress reduced the association between maternal abuse and attention problems. The associations between parental reported drinking and school adjustment were further reduced when controlling for the children's report of seeing their parents drunk, which itself predicted school adjustment. Controlling for parental mental distress did not reduce the associations.

**Conclusions:**

Parental alcohol abuse is an independent risk factor for attention and conduct problems at school. Some of the risk associated with mothers' drinking is likely to be mediated by adolescent mental distress. Despite lowered adjustment on the externalizing dimensions, children of alcohol abusers report that they enjoy being at school as much as other children.

## Background

Alcohol abuse and dependence are among the most prevalent psychiatric disorders [1,2], also among parents [3,4]. An extensive amount of research has been conducted on the psychological functioning of children of alcohol abusers, although relatively few studies have addressed these children's school adjustment. Most of the research on children of alcohol abusers recruits parents from clinical treatment or uses single responders for both exposure and outcome measures. This study investigates school adjustment, reported by a population based sample of adolescents, in relation to alcohol use reported by parents, while controlling for possible confounding or mediating psychosocial factors.

School adjustment can be defined as the degree to which adolescents "become comfortable, engaged and successful in their school environment" [5]. Previous research shows that compared to other children, children of alcohol abusers exhibit lower academic achievement [6,7]. This vulnerability is also reflected by their elevated risk for conduct problems, attention problems, hyperactivity, impulsiveness, delinquency, and unemployment [3,8-12]. Attention and conduct problems are important parts of school adjustment [13,14]. Dimensions such as satisfaction with school and academic performance would also be appropriate to include when assessing which types of school adjustment that may be impaired in children of alcohol abusers.

Theoretically, several perspectives predict impaired school adjustment and related psychopathology in these children. There is extensive evidence regarding the genetic influence on externalizing behaviour, and genetic co-variance between different kinds of externalizing behaviour [15-17]. Accordingly, one should expect children of alcohol abusers to have an increased probability of not only developing alcohol problems themselves, but also other kinds of externalizing behaviour. Prenatal alcohol exposure can also lead to poor academic performance [6,18]. Risk may also be transmitted by social strains linked to parental alcohol abuse, such as impaired parenting, or contextual factors, such as limited socioeconomic resources [19-23]. These burdens may make the children more susceptible to maladjustment, although each risk factor usually makes only small contributions to explaining variance in outcomes [24].

It is, however, difficult to isolate parental drinking from other risk factors. A part of the vulnerability seen among children of alcohol abusers may stem from other parental psychopathology, or from an accumulation of risk factors in the family. A majority of parents recruited through alcoholism treatment programmes had comorbid psychiatric problems [25]. Different studies have given conflicting results as to whether there is any remaining association between psychosocial functioning and parental alcohol abuse when controlling for other illnesses [25-28].

Moreover, findings from studies on abusers in treatment may not be generalizable to the general population. Only a small fraction of alcohol abusers in the general population are registered by clinics [4,8,29,30] and these are likely to have a more severe drinking problem, and more comorbid disorders [31]. Clinical studies may be well-suited for studying the children most affected by parental alcohol abuse, but less severe cases should be studied in population based samples [3,32]. However, studies with non-clinical assessment of alcohol abuse [33,34] often rely on single responders reporting on both their own outcome and, retrospectively, parental alcohol use. Response style and mood-congruent memory may lead to positive or negative responses to both measures, thereby yielding correlated error terms and inflated effect size estimates. Studies which leave the definition of alcohol abuse to the responder [32,35,36] are especially vulnerable to such biases.

Different effects of maternal and paternal alcoholism are understudied [12], although some studies suggest that maternal drinking has a greater impact than paternal drinking [8,37], or that maternal alcohol use is more predictive of internalizing problems, and paternal alcohol use of externalizing problems [25,38]. If maladjustment is transmitted by social strains, one should expect variables expressing stress to mediate the associations between parental drinking and child maladjustment. Therefore, if these children exhibit poor school adjustment, it is important to know whether this is caused by other problems they have previously been found to have, like mental distress [12,39] and poor social network [40,41], or whether it appears independent of those factors. A part of the causal chain may be exposure to parental drinking. One should expect that being directly exposed to parental drinking is more harmful than having parents who conceal their drinking. Moreover, as contextual factors may influence child adjustment, it is important to control for potential confounders, such as divorce, and other demographic variables.

The current study addresses methodological limitations in previous research by using a general population sample of adolescents and their parents to investigate four dimensions of school adjustment across the full range of parental drinking, from abstainers to abusers. By employing this method, high generalizability will be achieved. It was possible to study the unique contributions of maternal and paternal drinking and to control for parental mental distress as a possible confounder. Possible mediating effects of witnessing the parents intoxicated were investigated, and so were the possible mediation of effects of parental abuse on school adjustment by mental distress or poor social network.

## Methods

### Sample

The Nord-Trøndelag Health Study (HUNT-2) is a survey of the adolescent and adult population of Nord-Trøndelag County, Norway, carried out between 1995 and 1997. During school hours, 8984 adolescents (91% of the invited) aged 13 to 19 (mean age 16.0 years, SD = 1.8) filled in a questionnaire (Young-HUNT). Adolescents who were not enrolled in school (3%) were not invited.

At the same time, all inhabitants aged 20 or more were invited to the adult version of the survey, which consisted of a health examination and two questionnaires. The participating adolescents, of whom some were siblings, had a total of 7036 invited mothers (mean age 42.2 years, SD = 5.3), of which 71.9% replied to both questionnaires. Among 6535 invited fathers (mean age 45.2 years, SD = 5.7), 61.1% returned both questionnaires. More details regarding the HUNT-2 [42] and Young-HUNT [43] studies have been described elsewhere and are available at http://www.ntnu.edu/hunt.

### Ethics

The data matching between family members was carried out by Statistics Norway using personal birth identity numbers assigned to every Norwegian citizen. Before the data were returned to the researchers, the identity number was deleted, thus preventing identification of the participants. The Norwegian Data Inspectorate and the Regional Ethics Committee have approved of the study. All responders gave their written informed consent.

### Measures

#### School adjustment

School adjustment was measured with 14 items related to various experiences in school. The measure has been used in several studies and has been described elsewhere [44,45]. All items had four response options, ranging from "never" to "very often". An exploratory factor analysis using oblique rotation and polychoric correlations for ordinal data revealed that a solution with four factors provided a good fit (CFI = 0.99, TIL = 0.97, RMSEA = 0.05) and was psychologically meaningful. The factors were labelled attention problems ("attention"), satisfaction with academic results ("academic"), conduct problems ("conduct"), and dissatisfaction with school in general ("dissatisfaction"). Sum scores for each factor were calculated.

The items with the highest loadings on attention problems were "Become bored or dissatisfied", "Have difficulties concentrating during class" and "Skip school". Satisfaction with academic results was measured with "Understand what is being taught" and "Are satisfied with your test results". Conduct problems had the highest loadings from "Are reprimanded by the teacher", "Argue with the teacher", "Get in a fist fight", and "Cannot manage to be calm/sit still during class". Dissatisfaction with school in general consisted of "Look forward to going to school", "Think that gym or art is fun", "Think other classes are fun", and "Have fun during recess/break time". One question that did not fit into any factor was excluded ("Are teased/harassed by other students"). The Cronbach's alpha was 0.60 for the attention dimension, 0.59 for academic, 0.64 for conduct and 0.56 for dissatisfaction.

Due to a highly skewed distribution, the conduct problem score was natural logarithmically transformed to obtain a closer to normal distribution. All factors were scaled such that high values indicated poor adjustment and standardized in order to show effect sizes in terms of fractions of standard deviations.

#### Parental alcohol use

A combination of reported consumption and the CAGE alcohol screening questionnaire [46] was used to define alcohol use. The respondents were asked whether they were abstaining from alcohol, and, if not, asked to numerically state how many days they usually drank alcohol during one month, and how many units of beer, wine and liquor they usually drank over a two-week period. The frequency and amount were summed. The CAGE questionnaire consists of four yes/no statements related to alcohol use. Two items regarding criticism and guilt were collapsed. Both had to be endorsed to score one point. These items may reflect attitudes to drinking rather than problem drinking itself. In our data, these two items also turned out to be considerably less associated with consumption than the other CAGE items ("cut down" and "eye-opener"), each scored as one point. Abstainers were scored "no" or 0 on missing items.

Parents were classified into four different categories: "abstainers", "light drinkers", "at risk drinkers" and "alcohol abusers". Abstainers were categorized as a separate group since they differ in some respects from people with very low consumption [10,47]. Parents were classified as alcohol abusers if they were among the top 10% consumers within their gender, together with having scored at least 1 on the collapsed CAGE questionnaire. Parents who either had a positive score on the collapsed CAGE or who were among the top 10% consumers were coded into the "at risk" category. The remaining responders were categorized as light drinkers and used as reference group. This classification rendered 2.2% (135) of participating mothers as alcohol abusers and 12.8% (781) as being at risk, while 4.5% (219) of fathers were alcohol abusers and 16.5% (807) at risk. Mothers and fathers classified as abusers scored on average 16.8 and 26.7 on the summative index combining frequency and amount mentioned above, which is 3.4 and 3.9 times as high as the sample means. The test-retest reliability was measured among 8298 parents who participated both in the present study and in a similar study conducted 11 years earlier (HUNT-1). The polychoric correlation between the present alcohol measure and drinking frequency in the previous survey was 0.63. This indicates that alcohol use is relatively stable and reliably measured.

#### Seeing parents drunk

Adolescents were asked whether they had seen either of their parents drunk. Five response categories were possible, ranging from "never" to "a few times a week". It was not possible to distinguish between having seen the mother or father drunk. Sibling correlations show high reliability: The polychoric correlation was 0.57 in 1483 pairs of siblings and 0.68 in 96 pairs with less than one year of age difference.

#### Adolescent mental distress

Mental distress among adolescents was measured with SCL-5, which consists of five items measuring symptoms of anxiety and depression over the last two weeks. It correlates 0.92 [48] with the 25-item Hopkins Symptom Checklist [49], on which it is based. Previous studies have concluded that the measure has satisfactory validity and reliability [48,50]. Cronbach's alpha in the present study was 0.79. The recommended [50,51] cut-off (mean ≥ 2) rendered 15.6% of the adolescents as mentally distressed.

#### Parental mental distress

Symptoms of anxiety and depression were measured by 13 out of 14 items from the Hospital Anxiety and Depression Scale [52] and the seven-item CONOR Mental Health Index [53]. The Cronbach's alpha for a global summative mental health indicator, including nine anxiety items and eleven depression items was 0.91 for mothers and 0.90 for fathers. The top 10% of mothers and fathers were coded as mentally distressed. On average, distressed mothers and fathers scored 2.44 and 2.49 standard deviations above the mean of parents who were not categorized as mentally distressed.

#### Social network

The adolescents' number of close friends was obtained with a single question (four response categories ranging from "none" to "four or more") and used as an index of social network.

#### Demographics

The governmental statistics agency Statistics Norway provided demographic data on age, sex, education, income, and marital status. Education was grouped into five categories. The income of fathers and mothers was totalled to reflect family income. The age of parents and adolescents was used as continuous measures. Marital status was used together with the personal identification numbers of husbands and wives to determine whether the parents of a child were living together as a married or cohabiting couple. Dissolved relationships included divorcees, people who never lived together, unknown, and deceased parents.

### Missing data

Missing data were imputed instrument-wise, using the SPSS Missing Value Analysis (MVA), Expectation Maximization (EM), for respondents with valid data for at least half the items of each instrument.

Across responders, 0.6% of the item scores used to calculate school adjustment were imputed, while 2.1% of the adolescents had more than 50% blank school adjustment items and were omitted from the analyses. For adolescent mental distress, 0.4% of the records were imputed, leaving 2.0% with missing instrument scores. Maternal and paternal mental distress had 6.7% and 4.9% of the records imputed, respectively, leaving 0.9% of mothers and 0.7% of fathers who participated with missing instrument scores. Maternal and paternal alcohol consumption had 0.7% and 0.4% of the data imputed, leaving 4.6% and 4.5% with missing values. Analyses ran with and without imputed data provided similar results. Only results from imputed data are presented.

In order to prevent children with only one participating parent from being excluded from the analyses, missing on the parental alcohol and mental distress variables was coded into separate categories, thus providing results for children of non-responding parents as well. In addition, to keep children with unidentified or dead fathers (N = 100) and mothers (N = 9) in the analyses, the missing parents' age was estimated from the age of the co-parent. This treatment of the data permitted all adolescents with valid school data to be included in the final sample.

### Statistical analyses

Multivariate analysis of covariance was conducted in order to investigate group differences in the four school adjustment dimensions, with maternal and paternal alcohol use as the primary predictors. Generalized Estimating Equations was applied to adjust for statistical dependence between siblings.

Separate analyses were run with maternal and paternal alcohol use as single predictors in order to observe the unadjusted associations. Subsequently, conjoint analyses were run, in which the statistical effects of each of the parent's alcohol use were adjusted for the other parent's alcohol use, in addition to adjusting for the demographic variables.

Next, adjustments were made for potentially confounding or mediating variables. The design does not permit safe conclusions regarding the status of some of the predictors as confounders or mediators. Nonetheless, we *a priori *tentatively classified the covariates as confounding or mediating factors, based on their assumed temporal relation to alcohol abuse. Divorce and parental mental distress were considered likely confounders. Adolescent mental distress, seeing parents drunk, and adolescent social network were considered possible mediators, as they are likely to occur after the onset of parental alcohol abuse. To see what changes each variable caused to the model, adjustments were made stepwise, adding one variable at a time to the conjoint demography adjusted analysis.

Ultimately, all variables were entered into the model simultaneously, yielding estimates of the unique direct association between school adjustment and each predictor.

All possible interaction terms between parental alcohol use and the child's age or sex or the confounders and mediators mentioned above were tested in the model controlling for demography and both parent's alcohol use. The possible interaction effect between paternal and maternal alcohol use was also tested. In total 15 possible interactions were tested. Bonferroni adjustment would have suggested α = 0.003. This is, however, known to be too conservative and to reduce the power of the study [54], so to share trends with the reader, interaction effects with p < 0.01 are reported.

#### Software

The "polycor" library of R version 2.11.1 was used for calculating polychoric correlations. Mplus 5.2 was used to factor analyse the polychoric correlation matrix. Subsequent analyses were run in SPSS 17.0.

## Results

### Crude and partially adjusted associations

Correlations between the four dimensions of school adjustment varied between 0.11 and 0.41, with an average of 0.31. Table 1 presents crude group differences in the adolescents' school adjustment by their mothers' and fathers' alcohol use, as well as results adjusted for the other parent's alcohol use and for demographics. Since the outcome variables were standardized, results are given as group scores above or below the reference group in fractions of standard deviations, denoted *d *in the tables.

**Table 1:** Crude and adjusted associations between parental alcohol use and four dimensions of school adjustment

		Attention			Academic			Conduct			Dissatisfaction		
					
	N	d		**95% C.I**.	d		**95% C.I**.	d		**95% C.I**.	d		**95% C.I**.
**Crude associations**													
**Maternal alcohol**			***			***			***				
Abuse	134	0.35	**	0.13 - 0.56	0.03		-0.15 - 0.21	0.32	**	0.11 - 0.53	0.05		-0.14 - 0.24
At risk	768	0.15	***	0.07 - 0.23	0.02		-0.06 - 0.10	0.10	*	0.02 - 0.16	-0.02		-0.09 - 0.06
Abstainer	491	-0.11	*	-0.20 - -0.02	-0.12	*	-0.22 - -0.03	-0.19	***	-0.28 - -0.11	-0.01		-0.11 - 0.09
Missing response	2818	0.09	***	0.04 - 0.14	0.12	***	0.07 - 0.17	0.04	-	0.00 - 0.09	0.03		-0.02 - 0.08
Light drinking	4580	0			0			0			0		
**Paternal alcohol**			***			***			***			**	
Abuse	215	0.23	**	0.08 - 0.38	0.02		-0.13 - 0.17	0.24	**	0.08 - 0.40	0.08		-0.07 - 0.22
At risk	799	0.13	**	0.05 - 0.20	0.02		-0.06 - 0.10	0.14	***	0.06 - 0.21	0.04		-0.04 - 0.12
Abstainer	281	-0.08		-0.19 - 0.04	-0.23	***	-0.36 - -0.10	-0.07		-0.18 - 0.04	-0.08		-0.21 - 0.05
Missing response	4000	0.14	***	0.10 - 0.19	0.13	***	0.08 - 0.17	0.14	***	0.09 - 0.18	0.08	**	0.03 - 0.12
Light drinking	3496	0			0			0			0		
**Adjusted for other parent's alcohol use and for demography**													
**Maternal alcohol**			***			**			***				
Abuse	134	0.27	*	0.06 - 0.49	0.06		-0.11 - 0.24	0.27	**	0.07 - 0.48	0.04		-0.15 - 0.23
At risk	768	0.09	*	0.01 - 0.17	0.06		-0.02 - 0.14	0.08		0.00 - 0.16	-0.02		-0.09 - 0.06
Abstainer	491	-0.12	*	-0.22 - -0.02	-0.07		-0.18 - 0.04	-0.18	***	-0.28 - -0.08	0.02		-0.09 - 0.12
Missing response	2818	0.03		-0.02 - 0.08	0.05		0.00 - 0.10	-0.01		-0.06 - 0.04	-0.01		-0.06 - 0.04
Light drinking	4580	0			0			0			0		
**Paternal alcohol**			***						***				
Abuse	215	0.21	**	0.05 - 0.36	0.06		-0.09 - 0.20	0.18	*	0.01 - 0.34	0.10		-0.05 - 0.24
At risk	799	0.11	**	0.03 - 0.18	0.03		-0.05 - 0.11	0.11	**	0.03 - 0.19	0.05		-0.03 - 0.12
Abstainer	281	-0.04		-0.16 - 0.08	-0.17	*	-0.31 - -0.04	0.06		-0.06 - 0.19	-0.09		-0.24 - 0.05
Missing response	4000	0.09	***	0.04 - 0.14	0.06	*	0.01 - 0.11	0.11	***	0.06 - 0.16	0.05		-0.01 - 0.10
Light drinking	3496	0			0			0			0		

Demography includes adolescent age and sex, parental age, education and income.

Cohen's *d *express group differences as fractions of standard deviations.

* = p < 0.05; ** = p < 0.01; *** = p < 0.001.

Univariate results show that children of abusing and at-risk mothers and fathers had moderately higher levels of attention and conduct problems (upper part of Table 1). In particular, maternal problem drinking seems to be important for maladjustment in children. Children of abstaining mothers had lower levels of problems on the attention, academic and conduct dimensions in comparison to light drinkers, while abstaining fathers indicated better academic adjustment only.

The alcohol use of mothers and fathers was related, with a polychoric correlation of 0.58. When the two parents' drinking were entered into the model at the same time and adjusted for demographics, the associations with attention and conduct problems were somewhat reduced (lower part of Table 1). Parental alcohol use was still associated with their children's adjustment on attention and conduct problems, and children of abstainers still did better than children of light drinkers on attention and conduct if the mother was abstaining, and on academic if the father was abstaining. Children of mothers who did not participate did just as well as children of light drinkers, whereas children of non-responding fathers had modestly elevated scores on attention, academic, and conduct problems.

Since parental problem drinking was not associated with satisfaction with academic results or with school in general, further results for these outcome variables are not shown.

### Confounders and mediators

Each of the variables possibly confounding or mediating the associations between parental alcohol use and school adjustment were added to the conjoint adjusted analyses, one at a time.

When relationship dissolution, parental mental distress, or number of friends was added to the analyses, changes in associations between parental drinking and school adjustment were negligible, all changes Δd ≤ 0.02. Due to these small differences compared to the lower part of Table 1, the full results after entering each of these predictors are not tabulated at this stage. However, as these variables had independent associations with school adjustment, they are again included in the final analysis.

Results adjusted for adolescent mental distress and report of seeing parents drunk are shown in Table 2. The associations between maternal alcohol abuse and attention problems and conduct problems were weakened when the adolescents' level of mental distress was added to the analyses (Δd = 0.08 for attention, Δd = 0.03 for conduct). Associations with paternal alcohol abuse remained nearly unchanged (Δd = 0.01), for both attention and conduct problems. Changes in estimates for at-risk drinking were small (Δd ≤ 0.02). Although the estimates for maternal alcohol abuse fell below the significance level, maternal abuse was still as strong a predictor as paternal abuse.

**Table 2:** Results adjusting for adolescent mental distress and for seeing parents drunk

		Attention			Conduct		
			
	N	d		**95% C.I**.	d		**95% C.I**.
**Adjusted for adolescent mental distress**							
**Maternal alcohol**			***			***	
Abuse	133	0.19		-0.01 - 0.40	0.24	*	0.03 - 0.45
At risk	764	0.08	*	0.01 - 0.16	0.08		0.00 - 0.15
Abstainer	489	-0.09		-0.18 - 0.01	-0.16	**	-0.26 - -0.06
Missing response	2796	0.03		-0.02 - 0.08	-0.01		-0.06 - 0.04
Light drinking	4546	0			0		
**Paternal alcohol**			**			***	
Abuse	215	0.20	**	0.05 - 0.35	0.17	*	0.01 - 0.33
At risk	793	0.09	*	0.02 - 0.16	0.10	*	0.02 - 0.18
Abstainer	279	-0.06		-0.18 - 0.06	0.05		-0.08 - 0.18
Missing response	3963	0.07	**	0.02 - 0.12	0.10	***	0.05 - 0.15
Light drinking	3478	0			0		
**Adolescent mental distress**							
Distressed	1361	0.82	***	0.76 - 0.88	0.37	***	0.31 - 0.43
Not distressed	7367	0			0		
**Adjusted for seeing parents drunk**							
**Maternal alcohol**							
Abuse	131	0.20		-0.01 - 0.42	0.20		-0.01 - 0.42
At risk	755	0.05		-0.03 - 0.13	0.04		-0.04 - 0.12
Abstainer	477	-0.06		-0.16 - 0.04	-0.11	*	-0.21 - 0.00
Missing response	2742	0.02		-0.03 - 0.07	-0.02		-0.07 - 0.04
Light drinking	4490	0			0		
**Paternal alcohol**						**	
Abuse	209	0.14		-0.01 - 0.29	0.07		-0.09 - 0.23
At risk	781	0.05		-0.03 - 0.12	0.04		-0.04 - 0.12
Abstainer	273	0.03		-0.09 - 0.16	0.15	*	0.02 - 0.28
Missing response	3901	0.07	**	0.02 - 0.12	0.09	***	0.04 - 0.14
Light drinking	3431	0			0		
**Seen parents drunk**			***			***	
A few times a week	111	0.71	***	0.43 - 0.98	0.71	***	0.46 - 0.97
A few times a month	453	0.50	***	0.39 - 0.61	0.51	***	0.41 - 0.62
A few times a year	1746	0.25	***	0.19 - 0.31	0.36	***	0.30 - 0.42
A few times	3181	0.20	***	0.16 - 0.25	0.25	***	0.20 - 0.30
Never	3104	0			0		

Controlled for demography (adolescent age and sex; parental age, education and income).

Cohen's *d *express group differences as fractions of standard deviations.

* = p < 0.05; ** = p < 0.01; *** = p < 0.001.

When including the predictor variable "seeing parents drunk" in the analyses, all statistical effects of parental abuse or at-risk drinking were reduced to a non-significant size. The strongest reductions in effect size took place for paternal alcohol abuse and conduct problems. Both adolescents' mental distress and report of seeing their parents drunk were strongly predictive of school adjustment.

### All predictors combined

When all predictors were entered into the model at the same time, the estimates for maternal abuse decreased further, while those of paternal abuse were similar to the results from the analysis that included the variable of seeing parents drunk. Although parental drinking was not significantly associated with school adjustment, report of seeing parents drunk was predictive of maladjustment in school. Children of parents with dissolved relationships, and mentally distressed adolescents also had more conduct and attention problems. The father's mental distress predicted more attention problems, whereas the children who had a good social network scored higher on the conduct problems scale. All missing value groups deviated little (d values 0.01 - 0.07) from the reference groups. The results for all predictor variables except demography are shown in Table 3.

**Table 3:** Associations between parental alcohol use and attention and conduct problems, adjusting for all covariates

		Attention			Conduct		
			
	N	d		**95% C.I**.	d		**95% C.I**.
**Maternal alcohol**							
Abuse	130	0.13		-0.08 - 0.33	0.17		-0.04 - 0.39
At risk	748	0.04		-0.04 - 0.12	0.03		-0.05 - 0.11
Abstainer	474	-0.05		-0.15 - 0.04	-0.09		-0.19 - 0.01
Missing response	2714	0.02		-0.05 - 0.08	0.02		-0.05 - 0.08
Light drinking	4448	0			0		
**Paternal alcohol**							
Abuse	209	0.12		-0.03 - 0.28	0.05		-0.11 - 0.21
At risk	770	0.02		-0.05 - 0.10	0.04		-0.05 - 0.12
Abstainer	269	0.01		-0.11 - 0.13	0.14	*	0.01 - 0.27
Missing response	3861	0.04		-0.03 - 0.10	0.04		-0.02 - 0.11
Light drinking	3405	0			0		
**Adolescent mental distress**							
Distressed	1325	0.79	***	0.72 - 0.85	0.38	***	0.32 - 0.44
Not distressed	7189	0			0		
**Maternal mental distress**							
Distressed	677	0.06		-0.02 - 0.14	0.00		-0.08 - 0.08
Missing response	1658	0.01		-0.07 - 0.08	-0.07		-0.15 - 0.01
Not distressed	6198	0			0		
**Paternal mental distress**			*				
Distressed	559	0.11	**	0.03 - 0.20	0.06		-0.03 - 0.15
Missing response	2739	0.02		-0.05 - 0.09	0.06		-0.01 - 0.14
Not distressed	5237	0			0		
**Relationship dissolution**							
Dissolved	1841	0.14	***	0.08 - 0.20	0.09	**	0.03 - 0.15
Married or cohabiting	6673	0			0		
**Number of friends**						***	
None	138	0.18		-0.02 - 0.37	-0.34	***	-0.50 - -0.17
One	401	0.08		-0.02 - 0.18	-0.24	***	-0.34 - -0.14
Two or three	2735	-0.01		-0.06 - 0.03	-0.15	***	-0.20 - -0.11
Four or more	5240	0			0		
**Seen parents drunk**			***			***	
A few times a week	110	0.56	***	0.30 - 0.81	0.62	***	0.37 - 0.87
A few times a month	447	0.41	***	0.31 - 0.52	0.46	***	0.35 - 0.56
A few times a year	1736	0.20	***	0.15 - 0.26	0.33	***	0.27 - 0.39
A few times	3147	0.18	***	0.13 - 0.22	0.23	***	0.18 - 0.28
Never	3074	0			0		

Controlled for demography (adolescent age and sex; parental age, education and income).

Cohen's *d *express group differences as fractions of standard deviations.

* = p < 0.05; ** = p < 0.01; *** = p < 0.001.

### Interactions

No interaction effects statistically significant at the 0.01 level were found between maternal and paternal alcohol use or between alcohol use and parental mental distress or the child's gender, age, social network or mental distress. An interaction effect was found between paternal alcohol use and relationship dissolution on attention (Wald Type III = 14.766; p = 0.005). Children of alcohol abusing fathers with dissolved relationships had higher levels of attention problems than expected from the totalled main effects of abuse and relationship dissolution (additional effect: d = 0.57, C.I. 0.15 - 0.99, p = 0.007). The effect of seeing parents drunk varied with paternal alcohol use category on attention (Wald Type III = 181.93; p < 0.001) and conduct problems (Wald Type III = 81.41; p < 0.001). Post hoc tests included too many group combinations to provide meaningful results, but seeing parents drunk tended to be more predictive of these problems if the father did not participate or if the father was in the at risk group.

## Discussion

Maternal and paternal alcohol abuse or at-risk drinking was associated with moderately higher levels of attention and conduct problems, both at the crude level and when demography and the other parent's consumption was controlled for. There seems to be a dose-response trend, as the at-risk groups consistently scored between abusers and light drinkers on these outcomes. Heavy drinking in the parents did not predict dissatisfaction with school in general or with academic results in any of the analyses, even though this study has high power. Parental alcohol use predicted poor adjustment only on the impulse control-related dimensions attention and conduct. It is not surprising that we find associations with these dimensions. Children of alcoholics have previously been found to have elevated risks for attention and conduct problems [7,18] and the related diagnoses ADHD [15] and conduct disorder [16]. Previous studies show strong genetic components in the link between externalizing behaviour in parents and the children, such as parental drinking and behavioural control in the offspring [15,16]. Also, social strains such as negative life events, family conflict or dysfunction, disruption of routines, or neglect can also foster maladjustment [19,20]. From the social strain perspective, a lack of association between parental alcohol abuse and satisfaction at school could simply be explained by the school representing an escape from a troublesome home environment for some adolescents.

Maternal drinking was particularly predictive of high attention and conduct problem scores in our data. Our results are consistent with previous findings that maternal drinking has a greater impact on children than paternal drinking [8,37]. If not simply due to statistical fluctuations, the apparent heightened risk associated with mothers compared to fathers may be explained by impairment of the primary caregiver role, commonly undertaken by the mother, or by drinking during pregnancy. The present study does not have data on drinking during pregnancy, but previous studies have found that moderate prenatal exposure to alcohol increases the risk of conduct problems [10,18] and learning difficulties [6]. Also, since women drink less than men on average, pathological drinking in mothers may indicate a more severe stressor or higher heritable vulnerability to impulse control problems than drinking in fathers.

Children of abstainers had fewer attention, conduct, and academic problems than children of light drinkers. This finding stands in contrast to the results from a British study [10]. One may speculate that different factors lead to abstention in Norway and in the United Kingdom. As light drinking among parents is unlikely to constitute a social strain, we believe it is more likely that the difference between abstainers and light drinkers stems from lifestyle or personality factors rather than alcohol use per se.

Previous studies disagree on whether other parental psychopathology confounds the association between parental drinking and psychosocial functioning among their children [25-28]. The associations seen in the present study cannot be ascribed parental mental distress as it did not act as a confounder: adding this variable to the analysis did not substantially alter associations between parental drinking and school adjustment. Parental psychopathology may be more severe in studies finding such confounding. In addition, a high number of untreated cases in the general population, likely to be included in the present study, occur without severe comorbidity [1].

Adolescent mental distress was a strong predictor of attention problems, and a moderate predictor of conduct problems. Adolescent mental distress was moreover associated with maternal drinking, and the association between maternal abuse and attention problems was reduced when this variable was added to the analyses. It may therefore be considered a partial mediator for maternal alcohol use on attention problems. Before adjusting for adolescent mental distress, maternal drinking was more strongly associated with attention problems than was paternal drinking. Hence, it may be that maternal drinking has some additional effect on attention problems that is mediated by the adolescents' mental distress. However, most associations were independent of this distress: similar mediation was not seen between paternal drinking and attention problems, and the mediation on conduct seems to be small or non-existent.

The adolescent self-report of having witnessed parental drunkenness was a stronger predictor of maladjustment than was parental alcohol report. Adolescent report of seeing their parents drunk was associated with both parental report of drinking and outcome, and all effect sizes were reduced when this variable was added to the analyses. Hence, it is likely that seeing one's parents drunk mediates a non-trivial part of the association between parental alcohol use and school adjustment. One interpretation of this would be that being with intoxicated parents is harmful in itself, and that this question measures the subjective burden of having an alcoholic parent. Alternatively, this question may tap into variation in alcohol problems that is not captured by our parental alcohol measure. However, unlike with parent-reported measures, associations between predictors and outcome both reported by the adolescents may also partially reflect mood-congruent response consistency.

Children who had alcohol abusing fathers with a dissolved relationship were particularly at risk for attention problems. This may be an example of the principle that an accumulation of risk factors is especially harmful [25,55]. The risk seems to be equal across age and gender, as no interaction effects were found on these variables.

### Methodological considerations

As Young-HUNT data were collected during school hours, the adolescent sample is fairly representative of adolescents in the county, with most non-response resulting from sick leave. Parental response rates were lower. Although people who are struggling with many problems at once, or with very severe problems, seem to be underrepresented in population surveys [56], alcohol use only moderately predicts non-participation in the HUNT study [57]. In addition, simulations have shown that associations between variables are only moderately weakened by high rates of selective non-response [56]. We therefore believe that all consumption groups are adequately represented in the sample, and that it is suited for studying alcohol use within the general population.

Alcohol consumption is usually underreported in population studies [58]. If this underreporting changes the ranking of individuals, misclassification occurs. However, the alcohol consumption measure showed good reliability, with consistent scores over a long period (11 year test-retest correlation was 0.63). The prevalence of abuse in this study was also lower than usually reported [2], and due to the representativeness of the sample [57] and the strict inclusion criteria for the abuse groups, the large majority of people classified as abusers are likely to be true cases. False negatives, however, can lead to an underestimation of the number of exposed adolescents.

A strength in our study was that mothers and fathers reported their alcohol use and mental distress independently, thereby avoiding inflated effect sizes due to single responders reporting on all measures. There may, however, be correlated errors between measures reported by the same person. Since this was a general health study covering a large number of topics, respondents were not aware of the purpose of the alcohol questions, which has probably also reduced the risk of response bias.

We did not detect any confounding by comorbid parental disorders, perhaps because we only measured internalizing symptoms in the parents. It may be that parental externalizing behaviour or antisocial personality characteristics in reality confound or mediate the effects of parental alcohol abuse [7]. However, there were no data available on parental psychopathology besides of internalizing symptoms. In addition, as this study is cross-sectional, we cannot conclude on causal mechanisms or persistence of the problems. The inclusion of a missing category was necessary to avoid excluding many problem drinkers whose spouses did not participate. This implies a not fully complete control of mothers' and fathers' unique contributions to school adjustment.

## Conclusions

In spite of the mentioned limitations, we were able to study a representative sample of adolescent children of people with drinking problems, with independent reports from both parents and adolescents. More research is needed to investigate the specific effects of mothers' and fathers' drinking, causal mechanisms, reasons why child report of parental drinking appears to be more highly correlated with maladjustment than parental report, and factors that influence abstention.

Parental alcohol abuse is an independent risk factor for attention and conduct problems at school, which is not fully mediated by adolescent mental distress. While the association between parent-reported drinking and school adjustment seems to be modest when alcohol abuse occurs without comorbid disorders, witnessing the parents drunk was a stronger predictor for poor adjustment. The association between school adjustment and both parents' alcohol use seem to be mediated by seeing the parents drunk. We cannot exclude that direct exposure to drunken parents partially causes the problems. Maternal drinking may be worse for children than paternal drinking, and maternal drinking may have an effect partially mediated by adolescent mental distress. Only the externalizing dimensions were associated with parental alcohol abuse. Despite more attention and conduct problems, children of alcohol abusers enjoy school as much as other children.

## Competing interests

The authors declare that they have no competing interests.

## Authors' contributions

FAT is mainly responsible for the design, analyses and drafting of the manuscript. KR contributed to the methodological design and analyses. HA contributed to the analyses. ER supervised the methodology and performed the factor analysis. KT participated in designing the questionnaires, acquiring data, and contributed to the design and analyses. All authors read and approved the final manuscript.

## Pre-publication history

The pre-publication history for this paper can be accessed here:

http://www.biomedcentral.com/1471-2458/11/706/prepub
